# Impact of Brittle Creep Failure on Time-Delayed Characteristics of Rockburst

**DOI:** 10.3390/ma15093035

**Published:** 2022-04-22

**Authors:** Haozhe Chen, Zhushan Shao, Zhe Zhang

**Affiliations:** 1 School of Civil Engineering, Xi’an University of Architecture & Technology, Xi’an 710055, China; chenhaozhe515@foxmail.com (H.C.); zzhe0315@xauat.edu.cn (Z.Z.); 2 Shaanxi Key Lab of Geotechnical and Underground Space Engineering, Xi’an University of Architecture & Technology, Xi’an 710055, China; 3 School of Science, Xi’an University of Architecture & Technology, Xi’an 710055, China

**Keywords:** creep, brittle rock, time-delayed failure, excavation damage zone, rockburst

## Abstract

In this research, the combination of theoretical approach and numerical simulation was employed to comprehensively understand the initiation mechanism of time-delayed rockburst and analyze the time-delayed failure laws for surrounding rock after excavation unloading without prompt support. The investigations are principally at the angle of time and space, which refers to the creep property and damaged scope for surrounding rock. For the theoretical method, the analytical elastic and elastoplastic models for deep tunnel cross section and the creep model for brittle rock material from a microscopic view were combined. It was found that the time-delayed failure for surrounding rock resulted from the damage accumulation with crack development during the creep process. The surrounding rock with the elastic state was more stable than that in the plastic zone and the creep duration increased with growing distance from the center of tunnel section. Based on the theoretical creep model, the numerical simulation ulteriorly analyzed the brittle creep duration on the key positions. The surrounding rock tended to fail more in the strong excavation damage zone (SEDZ) than that in the weakly damaged zone (WEDZ), and brittle creep failure mainly occurred on the excavation border (EB) in a short space of time. In addition, the increase in the radius for tunnel cross section and the higher in situ stress distribution around the opening led to the acceleration of the creep process for surrounding rock, and the irregular cross-section shape of the tunnel caused the local damaged range extension and decreased the duration for time-delayed failure.

## 1. Introduction

When underground rock masses for excavation are subjected to high ground stress, the rockburst is likely to take place in a sudden or violent manner [1,2,3]. A typical and common phenomenon is that most rockburst events occur in a period of time after excavation unloading, which is in close connection with the creep failure of hard and brittle surrounding rock [4,5,6,7]. Although before the time-delayed rockburst, an obvious “peaceful period” accompanied by few microseismic activities can be monitored, the event may still occur within quite a short time and cannot be predicted in time, threatening the safety and stability during the underground construction [8,9].

The two dominating factors that affect the time-delayed failure laws for surrounding rock are the time-dependent duration and damaged range, corresponding to the aspects of time and space. The creep property of brittle rock material has been extensively studied, particularly the connection between the internal microscopic activity by cracks including initiation, propagation, and coalescence, and the rupture on the macro-scale, which is to understand the mechanism of brittle creep failure from various perspectives. Main (2000) proposed a damage mechanics model to describe the trimodal brittle creep behavior from a phenomenological perspective, considering the time-dependent subcritical crack growth [10]. Amitrano and Helmstetter (2006) and Fu et al. (2020) explored the time-dependent damage evolution inside brittle rocks on a mesoscopic scale by a numerical elastic-damage model and a discrete element grain-based stress corrosion model, respectively [11,12]. According to the microstructural observations through SEM and ultrasonic wave velocity surveys, Nicolas et al. (2017) discussed the effect of confining pressure on the deformation characteristics and failure mode through creep tests on limestone [13]. From the angle of space, the concept of excavation damage zone (EDZ) is chiefly defined by the multiple empirical criteria or inspection technology such as sonic wave testing and borehole camera [14,15,16,17], and EDZ was employed to different contexts for sites and projects. One of the most practical definitions in rock engineering is that the plastic scope for the surrounding rock around the excavation opening can be identified as EDZ, referring to the irreversible damage deformation to the surrounding rock [18,19,20].

Most research on deep rockburst behavior is primarily carried out through field monitoring and indoor experiment that are able to further assess and predict the stability of the rock mass environment to a large extent. Zhang et al. (2014) verified the quantificational relationship between rockburst proneness index and accumulative AE counts by conducting uniaxial compression tests accompanying AE technology on various brittle rock materials [21]. Zhou et al. (2018) established an AE-based rockburst criterion by monitoring the rockburst events in the Gaolilongshan tunnel reflected by real-time AE activities [22]. Dou et al. (2018) achieved the early warning of rockburst on the basis of comprehensive analysis through the MS (microseismic) multi-parameter index system and Liu et al. (2021) completed the real-time dynamic estimation on rockburst occurrence during tunnel excavation based on the proposed microseismicity-based method [23,24]. By using a micro-camera in a video monitoring system, Gong et al. (2018) observed the damage process of the circular hole sidewall in cubic rock samples in real-time under a true-triaxial loading condition, and analyzed the destructive features of rockburst caused by spalling damage [25].

However, the initiation mechanism of time-delayed rockburst mostly induced by the creep failure of surrounding rock in deep tunnel has been of relatively less focus [5,26], especially from the point of view of theory on the microscopic level. Therefore, in this study, on the basis of time-delayed rockbursts that occurred in the underground tunnels of the Jinping II hydropower station located in China, the time-delayed mechanism of rockburst in deep tunnel after excavation unloading without support and the laws on time-delayed failure duration and the damaged area of surrounding rock were investigated by combining the theoretical method and numerical simulation. The duration for brittle creep failure of surrounding rock at the key locations in the EDZ was analyzed, and the influences of high crustal stress at depth, opening radius, and shape of tunnel cross section on time-delayed failure laws for surrounding rock in EDZ are discussed.

## 2. Theoretical Models

### 2.1. Analytic Model for Deep Tunnel Section

#### 2.1.1. Elastic Analytic Model for Deep Tunnel Section

**Figure 1:**
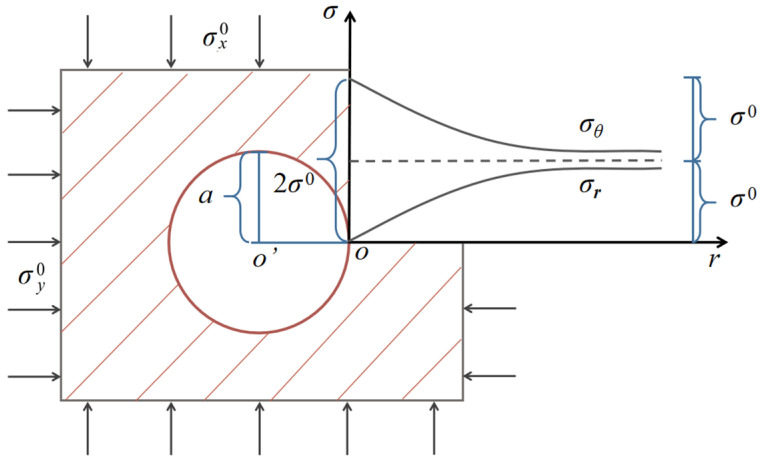
Elastic stress distribution for the surrounding rock of the deep tunnel cross section.

The cross section with a circular shape was set for the deep and infinitely long tunnel (Figure 1). It was assumed that the surrounding rock property stayed constant. The initial stress field was homogeneous, with a positive compressive state. The initial principal stresses $\sigma_{x}^{0}$, $\sigma_{y}^{0}$ are symmetrical to the vertical and horizontal axes of the tunnel cross section, respectively. In a state of plane strain, the elastic analytic expression for surrounding rock after excavation and support can be described by Xiang (2014) [27]:

(1) $$\left.\begin{array}{l} \sigma_{r}=F_{s}\frac{a_{}^{2}}{r_{}^{2}}+\frac{1}{2}\left(\sigma_{x}^{0}+\sigma_{y}^{0}\right)\left(1-\frac{a_{}^{2}}{r_{}^{2}}\right)+\frac{1}{2}\left(\sigma_{x}^{0}-\sigma_{y}^{0}\right)\left(1-4\frac{a_{}^{2}}{r_{}^{2}}+3\frac{a_{}^{4}}{r_{}^{4}}\right)cos2\theta \\ \sigma_{\theta }=-F_{s}\frac{a_{}^{2}}{r_{}^{2}}+\frac{1}{2}\left(\sigma_{x}^{0}+\sigma_{y}^{0}\right)\left(1+\frac{a_{}^{2}}{r_{}^{2}}\right)-\frac{1}{2}\left(\sigma_{x}^{0}-\sigma_{y}^{0}\right)\left(1+3\frac{a_{}^{4}}{r_{}^{4}}\right)cos2\theta \\ \tau_{r\theta }=-\frac{1}{2}\left(\sigma_{x}^{0}-\sigma_{y}^{0}\right)\left(1+2\frac{a_{}^{2}}{r_{}^{2}}-3\frac{a_{}^{4}}{r_{}^{4}}\right)sin2\theta \end{array}\right\}$$

where *a* is the radius of cross section and *r* is the distance from the center of excavation section; *σ_r_*, *σ_θ_* represent the radial and circumferential stresses for surrounding rock; and *F*_s_ is the uniform radial resistance of support to the surrounding rock.

Equation (1) is simplified as follows when the homogeneous initial stress field is considered isotropic and no support is used for the surrounding rock around the excavation opening ($\sigma_{x}^{0}$ = $\sigma_{y}^{0}$ = *σ*^0^, *τ_rθ_* = 0 and *F*_s_ = 0):

(2) $$\left.\begin{array}{l} \sigma_{r}=\sigma_{}^{0}\left(1-\frac{a_{}^{2}}{r_{}^{2}}\right)\\ \sigma_{\theta }=\sigma_{}^{0}\left(1+\frac{a_{}^{2}}{r_{}^{2}}\right) \end{array}\right\}$$

#### 2.1.2. Elastoplastic Analytic Model for Deep Tunnel Section

It should be noted that the active surrounding rock pressure was employed in all cases, which means that *F*_s_ < *σ*^0^. When *a* ≤ *r* ≤ *a*_p_ (*a*_p_ is the radius of plastic zone), the radial and circumferential stresses ($\sigma_{r}^{p}$ and $\sigma_{\theta }^{p}$) for the surrounding rock with the plastic status can be expressed as [27]:

(3) $$\left.\begin{array}{l} \sigma_{r}^{p}=\left(F_{s}+\frac{c}{tan\varphi }\right){\left(\frac{r}{a}\right)}^{\frac{2sin\varphi }{1-sin\varphi }}-\frac{c}{tan\varphi }\\ \sigma_{\theta }^{p}=\left(F_{s}+\frac{c}{tan\varphi }\right)\left(\frac{1+sin\varphi }{1-sin\varphi }\right){\left(\frac{r}{a}\right)}^{\frac{2sin\varphi }{1-sin\varphi }}-\frac{c}{tan\varphi } \end{array}\right\}$$

where *φ* and *c* correspond to the internal friction angle and cohesion of the surrounding rock material.

For the surrounding rock in elastic zone (*r* ≥ *a*_p_), the radial and circumferential stresses are given by:

(4) $$\left.\begin{array}{l} \sigma_{r}^{e}=\sigma_{}^{0}-{\left(\frac{a_{p}}{r}\right)}^{2}\left(\sigma_{}^{0}-\frac{2\sigma_{}^{0}-\sigma_{c}}{\xi +1}\right)\\ \sigma_{\theta }^{e}=\sigma_{}^{0}+{\left(\frac{a_{p}}{r}\right)}^{2}\left(\sigma_{}^{0}-\frac{2\sigma_{}^{0}-\sigma_{c}}{\xi +1}\right) \end{array}\right\}$$

where *ξ* = (1 + sin *φ*) / (1 − sin *φ*) and *σ*_c_ is the short-term strength of the rock material.

#### 2.1.3. Stress State on Key Positions

It is shown in Figure 1 that when *r* = *a*, referring to the excavation surface in a deep tunnel, the elastic radial and circumferential stress distribution for the surrounding rock is obtained: *σ_r_* = 0, *σ_θ_* = 2*σ*^0^. Both *σ_r_* and *σ_θ_* gradually approach *σ*^0^ with increasing *r*.

**Figure 2:**
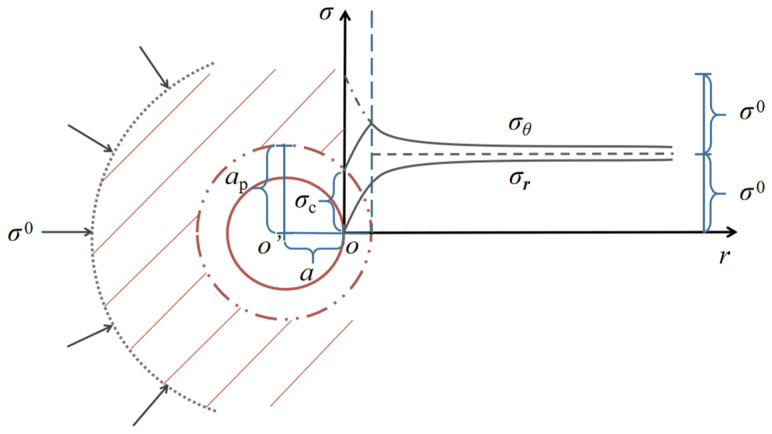
Elastoplastic stress distribution for the surrounding rock of the deep tunnel cross section.

The initial stress field around the opening in the elastoplastic condition is exhibited in Figure 2. For the case that *r* equals *a*, the radial and circumferential stresses that are distributed on the excavation border are achieved as: $\sigma_{r}^{p}$ = 0, $\sigma_{\theta }^{p}$ = *σ*_c_. At the junction of plastic and elastic areas (*r* = *a*_p_), the corresponding stress distribution is derived by:

(5) $$\left.\begin{array}{l} \sigma_{r}^{e}=\frac{2\sigma_{}^{0}-\sigma_{c}}{\xi +1}\\ \sigma_{\theta }^{e}=2\sigma_{}^{0}-\frac{2\sigma_{}^{0}-\sigma_{c}}{\xi +1} \end{array}\right\}$$

The variation for stress distribution in the elastic zone is similar to that in the elastic analytical model when *r* heads for infinite.

Hence, on the basis of the relation between maximum and minimum principal stresses in the polar coordinate system, which is derived as: *σ*_1_ − *σ*_3_ = 2{[(*σ_r_ − σ_θ_*) / 2]^2^ + *τ*^2^*_rθ_*}^1/2^ [28], the stress states on the rock surrounding the excavation surface under the elastic and elastoplastic conditions are (2*σ*^0^, 0) and (*σ*_c_, 0), respectively. The applied stress level on the plastic zone boundary is expressed by: *σ*_1_ = 2*σ*^0^ − [(2*σ*^0^ − *σ*_c_) / (*ξ* + 1)], *σ*_3_ = (2*σ*^0^ − *σ*_c_) / (*ξ* + 1).

### 2.2. Creep Model

#### 2.2.1. Micromechanical Model

**Figure 3:**
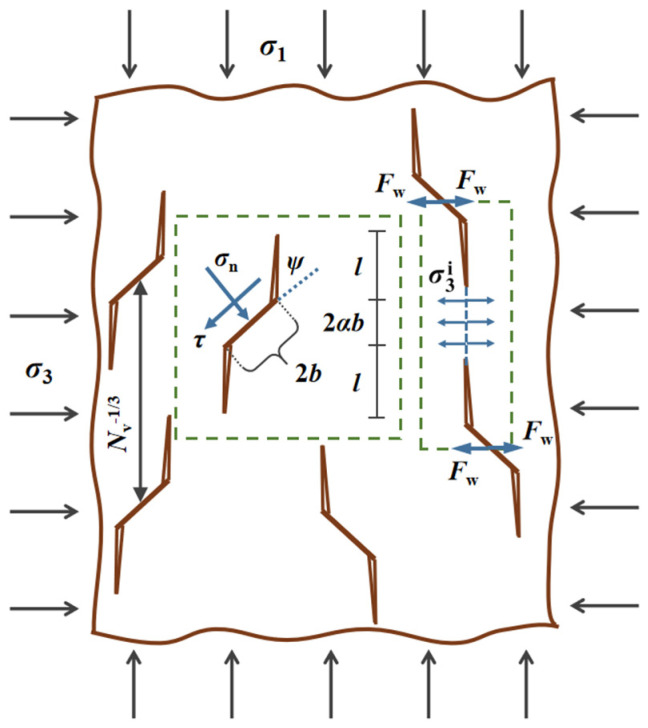
Schematic of the micromechanical model.

Figure 3 shows the micromechanical model with original cracks in the triaxial compressive state (*σ*_2_ and *σ*_3_ are equal) under three-dimensional condition. The rock material is regarded as an isotropic elastic body, containing penny-shaped microcracks with a radius *b*, and an angle *ψ* is set between the maximum principal stress *σ*_1_ and each initial crack. The wing crack with length *l* emerges at both original crack ends, lying parallel to the *σ*_1_ direction during the crack growth.

To characterize the initial crack density of rocks, the original damage was defined as: *D*_0_ = 4π*N*_v_(*αb*)^3^/3 [29], in which the number of microcracks per unit volume is denoted by *N*_v_ and *α* represents the projection of crack radius in the vertical plane, equaling to cos *ψ*.

A shear stress *τ* and a normal stress *σ*_n_ on the crack plane are created by the remote stress field (*σ*_1_, *σ*_3_), which is expressed as follows:

(6) $$\left.\begin{array}{l} \tau =\frac{\sigma_{3}-\sigma_{1}}{2}sin2\psi \\ \sigma_{n}=\frac{\sigma_{1}+\sigma_{3}}{2}-\frac{\sigma_{1}-\sigma_{3}}{2}cos2\psi \end{array}\right\}$$

The elastic strain initially drives the sliding between the crack interface without further crack extension when the static friction is exceeded by the shear stress *τ*, and the reverse takes place if |*τ*| ≤ *μ*|*σ*_n_|at the low stress level. The initiation of wing cracks at a critical stress *σ*_1c_ is described as [30]:

(7) $$\sigma_{1c}=\frac{\sqrt{1+\mu_{}^{2}}+\mu }{\sqrt{1+\mu_{}^{2}}-\mu }\sigma_{3}-\frac{\sqrt{3}}{\sqrt{1+\mu_{}^{2}}-\mu }\frac{K_{IC}}{\sqrt{\pi b}}$$

where *K*_ΙC_ is the fracture toughness of the mode I crack, also called the critical stress intensity factor; and *μ* is a friction coefficient that controls the sliding on the initial microcrack interface.

The mode I stress intensity factor at the wing crack tip is expressed by [29]:

(8) $$K_{I}=\frac{F_{w}}{{\left[\pi \left(l+\beta b\right)\right]}^{3/2}}+\frac{2}{\pi }\left(\sigma_{3}+\sigma_{{}_{3}}^{i}\right)\sqrt{\pi l}$$

where the wedging force being denoted by *F*_w_ is given by:

(9) $$F_{w}=\left(\tau +\mu \sigma_{n}\right)\pi b_{}^{2}sin\psi =-\left(A_{1}\sigma_{1}-A_{2}\sigma_{3}\right)b_{}^{2}$$

and

(10) $$\left.\begin{array}{l} A_{1}=\pi \sqrt{\frac{\beta }{3}}\left(\sqrt{1+\mu_{}^{2}}-\mu \right)\\ A_{2}=A_{1}\frac{\sqrt{1+\mu_{}^{2}}+\mu }{\sqrt{1+\mu_{}^{2}}-\mu } \end{array}\right\}$$

Furthermore, *F*_w_ is balanced by the internal stress $\sigma_{{}_{3}}^{i}$, that is:

(11) $$\sigma_{{}_{3}}^{i}=\frac{F_{w}}{\pi_{}^{1/3}{\left[3/\left(4N_{v}\right)\right]}^{2/3}-\pi {\left(l+\alpha b\right)}^{2}}$$

where *β* is a constant that blocks *K*_Ι_ from becoming infinite when *l* = 0. Thus, the stress intensity factor concerning the crack length *l* can be derived as [31]:

(12) $$\frac{K_{I}}{\sqrt{\pi b}}=-\left(A_{1}\sigma_{1}-A_{2}\sigma_{3}\right)\left(c_{1}+c_{2}\right)+\sigma_{3}c_{3}$$

where

(13) $$\left.\begin{array}{l} c_{1}=\frac{1}{\pi_{}^{2}}{\left(\frac{l}{b}+\beta \right)}^{-3/2}\\ c_{2}=2\frac{1}{{\left(\pi \alpha \right)}^{2}}{\left(\frac{l}{b}\right)}^{1/2}/\left[D_{0}{}^{-2/3}-{\left(1+\frac{l}{\alpha b}\right)}^{2}\right]\\ c_{3}=\frac{2}{\pi }{\left(\frac{l}{b}\right)}^{1/2} \end{array}\right\}$$

Without regard to the stress corrosion, the crack growth only occurs when *K*_Ι_ meets the fracture toughness *K*_ΙC_, and the relationship between the wing crack length *l* and the remote stress state (*σ*_1_, *σ*_3_) can be expressed as:

(14) $$\sigma_{1}\left(l\right)=\frac{\sigma_{3}\left[c_{3}\left(l\right)+A_{2}\left(c_{1}\left(l\right)+c_{2}\left(l\right)\right)\right]-K_{IC}/\sqrt{\pi b}}{A_{1}\left[c_{1}\left(l\right)+c_{2}\left(l\right)\right]}$$

The short-term strength of rock is equal to the maximum *σ*_peak__1_ of the function *σ*_1_(*l*). The corresponding equilibrium crack length *l*_0_ is obtained by Equation (14), taken as the initial length of microcrack for the numerical calculation during creep at the constant stress level (*σ*_1_, *σ*_3_), which is related to the crack initiation.

It has been found that under the action of stress corrosion, the sub-critical crack growth initiates when *K*_Ι_ < *K*_ΙC_. One of the empirical relationships between microcrack growth rate and stress intensity factor can be well used for a single mode I microcrack [32]:

(15) $$\frac{dl}{dt}=v{\left(\frac{K_{I}}{K_{IC}}\right)}^{n}$$

where *v* corresponds to the characteristic crack speed and *n* is an empirical exponent commonly called the stress corrosion index.

Hence, the expression that relates the wing crack length over time can be derived as follows:

(16) $$\frac{dl}{dt}=v{\left(\pi b\right)}_{}^{n/2}{\left[\frac{\left(A_{2}\sigma_{3}-A_{1}\sigma_{1}\right)\left(c_{1}+c_{2}\right)+\sigma_{3}c_{3}}{K_{IC}}\right]}^{n}$$

#### 2.2.2. Strain vs. Time

Considering that the numerous microcracks’ distribution is random, the Weibull probability distribution is used to describe the damage extent of rock [33,34]:

(17) $$D=1-exp\left[-{\left(\varepsilon /\varepsilon_{0}\right)}^{m}\right]$$

where *m*, *ε*_0_ are both the material constants. The elastic strain before the wing crack growth can be obtained:

(18) $$\varepsilon_{e}=\varepsilon_{0}{\left[-ln\left(1-D_{0}\right)\right]}^{1/m}$$

The relationship between the internal damage of rock and microcrack length is defined as [29]:

(19) $$D=\frac{4}{3}\pi N_{v}{\left(l+\alpha b\right)}^{3}$$

In the end, the connection between strain and crack growth length after combining the expressions (17) and (19) on the macro- and micro-scales is given as:

(20) $$\varepsilon =\varepsilon_{0}{\left\{-ln\left[1-\frac{{\left(l+\alpha b\right)}^{3}D_{0}}{{\left(\alpha b\right)}^{3}}\right]\right\}}^{1/m}$$

The time-dependent evolution of strain can be ulteriorly attained in accordance with the relation between microcrack length and time:

(21) $$\varepsilon =\varepsilon_{0}{\left\{-ln\left[1-\frac{{\left[l\left(t\right)+\alpha b\right]}^{3}D_{0}}{{\left(\alpha b\right)}^{3}}\right]\right\}}^{1/m}$$

The above creep model builds the bridge between the strain and microcrack growth through the expressions of internal damage reflected by the macroscopic and microscopic scales, and accounts for crack interactions from a different view by adjusting the effect of confining pressure with the growing axial cracks, which simultaneously takes the friction between the original microcrack surfaces into consideration.

### 2.3. Mechanical Properties on Macro- and Micro-Scales

**Figure 4:**
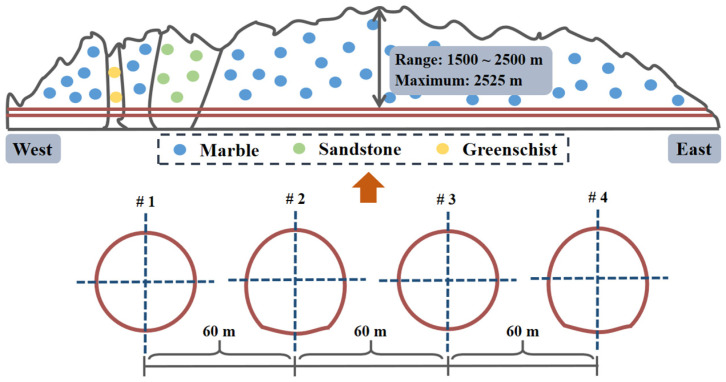
Schematic of the headrace tunnel for the Jinping II hydropower station.

In the following analysis, it was assumed that the cross section of all four parallel headrace tunnels for the Jinping II hydropower station were round in shape (Figure 4) [9]. In order to depict the homogeneous and isotropic initial stress field around the excavation opening, *σ*^0^ was set to be equivalent to the maximum (57 MPa) based on the field test for ground stress at the depth of 1900 m [35].

Given the high-risk area concerning rockburst due to the high natural stress and the geological environment along the tunnel, the mechanical properties of marble, that is, hard, brittle, and compact, were used for the theoretical models, which are listed in Table 1. The parameters on a macroscopic scale that contains the short-term strength, elastic modulus, etc. were obtained via the compression test [9,36]. At the micro level, the initial damage and the fracture toughness, index of stress corrosion, and characteristic crack velocity were separately derived from the results of scanning electron microscope test and subcritical crack growth test [37,38]. Combining the empirical relation of the crack initiation stress state (*σ*_1_ = 2.67*σ*_3_ + 46) [39] and Equation (7), the initial crack size and friction coefficient were obtained. The microcrack angle is usually assumed to be 45° considering the maximum shear impact [29,31]. The constant *β* was achieved by comparison between Equations (7) and (14) (*l* = 0), and the material constants *m* and *ε*_0_ were applied referring to the selection by Li and Shao (2016) [40]. The initial equilibrium crack length was calculated by submitting the stress state (46, 0) into Equation (14) (*l* > 0).

## 3. Time-Dependent Evolution of Crack Length and Strain

**Figure 5:**
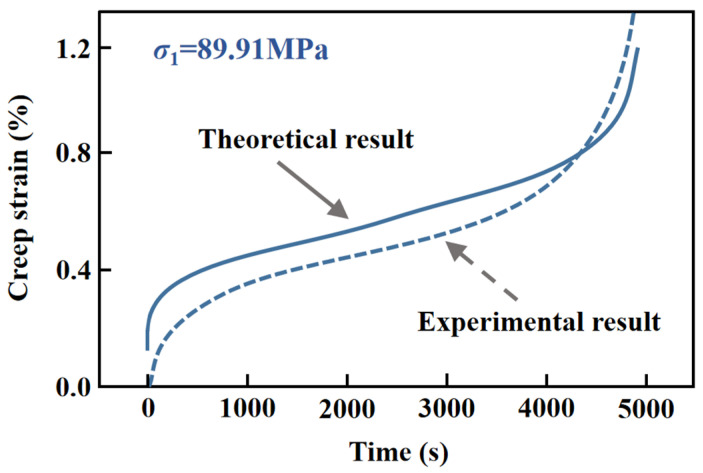
Comparison between the theoretical and experimental results of Sanxia granite [41].

**Figure 6:**
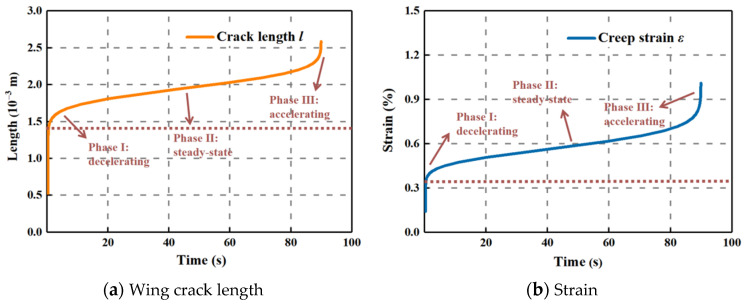
Time-dependent evolution of the microcracks’ length (**a**) and strain (**b**) at 114 MPa.

It is known that the creep model brings into proper correspondence with the existing experimental results on different brittle rocks (Figure 5) [40,41]. According to the stress distribution (2*σ*^0^, 0) on the excavation edge in the elastic analytical model for deep tunnel cross section and the mechanical property of Jinping marble, the variations in crack growth length and strain over time under the constant stress level of 114 MPa are exhibited in Figure 6a,b, respectively.

The wing crack length presented almost the same performance as the strain during the evolution process, which was composed of the three main segments: attenuating, steady-state, and accelerating stage, sequentially denoting the primary, secondary, and tertiary creep. Furthermore, the instantaneous elastic deformation rapidly emerged after loading and then entered into the primary creep phase.

The duration from initiation of deformation to failure for the surrounding rock was clearly short, which continued for approximately 90 s. The gradual internal damage accumulation was caused by the generation of new cracks and evolution of original cracks, dominating the continuous deformation of rock. When the density of cracks breaks the marginal level, the accelerating creep is triggered, during which the cracks extensively propagate and coalesce in a brief moment until the macroscopic failure plane appears, leading to a sudden rupture.

**Figure 7:**
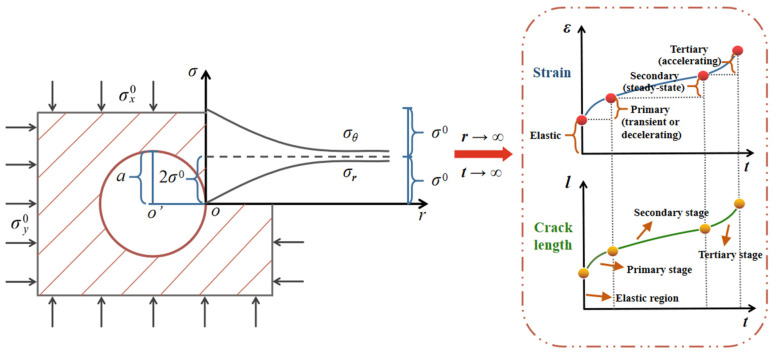
Time-dependent evolution of crack length and strain with distance from the center of tunnel section in the elastic analytic model.

Both the wing crack length and strain exhibited the parallel evolution laws and time-dependent behaviors, showing the intuitive connection between the micro- and macro-levels (Figure 6 and Figure 7). Figure 7 also shows that the duration for brittle creep failure gradually approached infinite as the distance *r* grew from the center of excavation section, which indicates that great stability for surrounding rock can be attained if the distance is long enough.

## 4. Time-Delayed Failure for Surrounding Rock in Deep Tunnel

In accordance with the on-site monitoring record on the underground tunnels for the Jinping II hydropower station, the frequent time-delayed rockbursts occurred in the headrace tunnels after excavation unloading with support and the delayed duration on the reported events ranged from six to 30 days, which were mostly linked to the surrounding rock regions formed by marble material [8,9]. Moreover, the relevant statistics also showed that most time-delayed rockburst events commenced in a couple of hours to several days for the auxiliary and testing tunnels [9,26]. Therefore, in the following discussion, it can be further assumed and speculated that the brittle creep failure for surrounding rock in a deep tunnel will likely take place within a distinctly brief period after excavation unloading if the support for the surrounding rock is not prompt, particularly under the high field stress condition.

### 4.1. Theoretical Illustration

**Figure 8:**
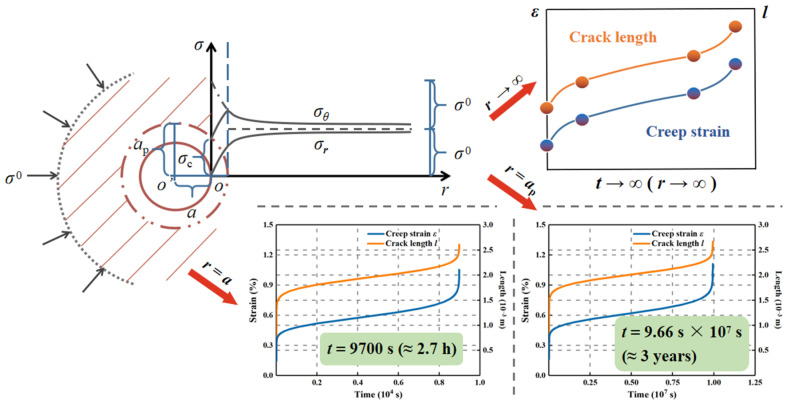
Time-dependent evolution of crack length and strain with distance from the center of tunnel section in the elastoplastic analytical model.

Combining the analysis in Section 3, Figure 8 further shows the time-dependent evolution of microcrack length and strain for the surrounding rock and corresponding time-delayed failure duration with distance *r* from the center of excavation section of a deep tunnel in the elastoplastic analytic model. When *r* = *a*_p_, the creep process continued for about 9.66 × 10^7^ s (three years), which far outweighed the case where *r* was equal to *a*. The rock mass environment basically remained steady (*t* = ∞) in the condition of *r* reaching infinite.

The above results suggest that the deformation of the surrounding rock takes on a strong time-dependent effect after excavation unloading without support. The rock surrounding the opening edge was most damaged and rapidly toward the creep failure, which was likely to fail in 2.7 h.

When *r* ≥ *a*_p_, the plastic zone around the excavation opening transitioned to the elastic zone, until the stress field was eventually in equilibrium by the stress state that *σ*_1_ = *σ*_3_ = *σ*^0^ with increasing distance *r*. Despite the more distinct time delay at the junction of plastic and elastic areas, the surrounding rock with the plastic status exhibited more instability than that in the elastic state. It was also observed that the duration for the creep process on the excavation edge is greatly determined by the short-term strength or uniaxial compressive strength of the surrounding rock, and both the short-term strength and initial stress field around the tunnel heavily impact the creep duration for the surrounding rock on the plastic zone border.

### 4.2. Numerical Illustration

Based on the above analysis, the numerical simulation model was built by MIDAS GTS NX, with dimensions of 100 m × 100 m and quadrilateral partition was used for the mesh. To acquire the more accurate applied stress state on the surrounding rock, the area around the tunnel excavation was divided into grid densification. The horizontal displacements were fixed on the left and right sides of the model, and the horizontal and vertical displacements were fixed on the lower side. The Mohr–Coulomb criterion was adopted for constitutive relation. For the round headrace tunnel section, the radii were set as 4, 6.5, and 10 m, and the arched structural geometry to test the tunnel cross section was 7.5 m × 8 m. The group with the maximum difference of geostresses was employed to simulate the most negative condition, and the stress fields at the depth of 1900 m and 2400 m corresponded to the headrace and testing tunnel models, respectively [9,35].

**Figure 9:**
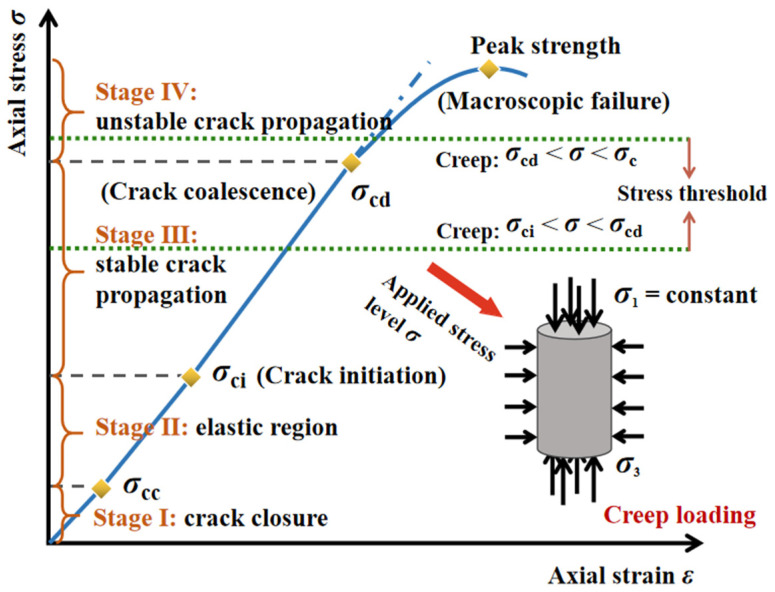
Schematic of crack development at stress thresholds.

According to the stress redistribution for the surrounding rock after excavation unloading and the stress thresholds of brittle rock material, a judgement criterion regarding the damaged zone range can be described by: *σ*_1_ − *σ*_3_ = *Aσ*_c_, *σ*_1_ − *σ*_3_ = *Bσ*_c_ [42]. Both *A* and *B* are material constants, and the scopes of *A* and *B* were 0.4~0.6 and 0.8~1.0, corresponding to the crack initiation and coalescence in the interior of the rock material. The stress thresholds of *σ*_ci_ (crack initiation) and *σ*_cd_ (crack coalescence) mark the commence of the stable and unstable stage during crack development (Figure 9) [43].

**Figure 10:**
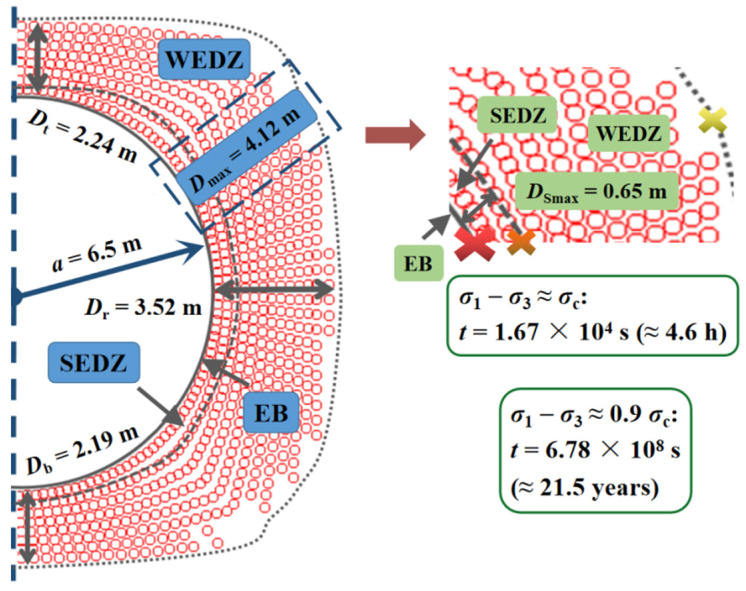
Time-delayed failure laws in EDZ for the round tunnel cross section at the depth of 1900 m (*a* = 6.5 m).

In the model, the plastic status area was considered as the excavation damage zone (EDZ), which was further divided into the strongly damaged zone (SEDZ) and weakly damaged zone (WEDZ), and the right half of the tunnel cross section was selected for analysis on account of the nearly symmetric results (Figure 10). Due to the creep process lasting long enough, the area near and outside the WEDZ can be deemed steady. For the more clear time-delayed failure laws on the surrounding rock after excavation unloading without support, *B* was set as 0.9 or 1 in the model, which refers to the unstable or even failure region in the SEDZ, covering the excavation border (EB) and the interface between the SEDZ and WEDZ. Next, the delayed failure duration for the surrounding rock on these critical locations can be achieved by substituting the corresponding stress state from the numerical results into the theoretical creep model (Section 2.2).

As presented in Figure 10, when the excavation radius was 6.5 m, the damage zone in the upper right reached the maximum (4.12 m) and the scope of SEDZ was 0.65 m. The surrounding rock that is close to the EB may fail in 4.6 h on the condition that *B* = 1 and the creep duration on the SEDZ boundary was about 21.5 years, showing an increasing trend for the time-delayed failure duration in the SEDZ. The area near or inside SEDZ is at high stake, resulting from the highly irreversible damage accumulation of the surrounding rock with continuous crack coalescence, which further forms the failure planes.

The apparent difference for creep duration on either border in SEDZ can also be observed in Figure 10. The surrounding rock near EB was damaged to a great degree after excavation unloading without support. Because of the stress adjustment, the fissure development, which comprises the generation, propagation, and the sliding along the structure plane, rapidly proceeds, bringing about the rupture from the excavation surface in only a few hours. In the meantime, the area close to the SEDZ border or inside the WEDZ presents more stability with slighter damage. However, in more severe cases, a larger opening section is shaped by spalling or even ejection from the rock surrounding the excavation surface during the first rockburst, which may cause creep failure in a wider original stable range of SEDZ within a shorter time. Thus, secondary rockburst may occur without sufficient and timely support for the failure zone after the first event.

**Figure 11:**
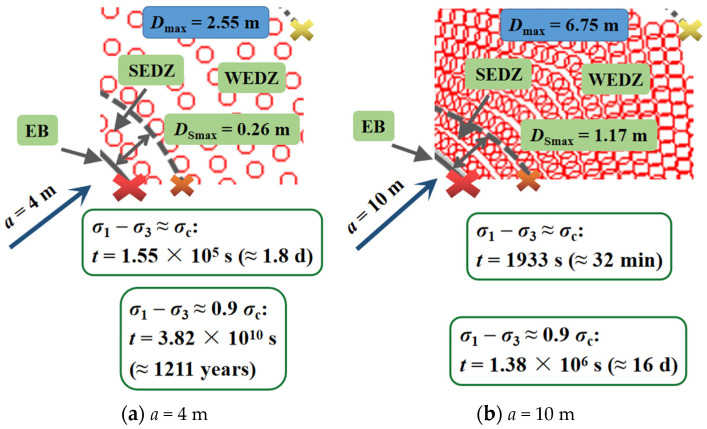
Time-delayed failure laws in the EDZ for the round tunnel cross section at the depth of 1900 m (*a* = 4 m (**a**) and 10 m (**b**)).

**Figure 12:**
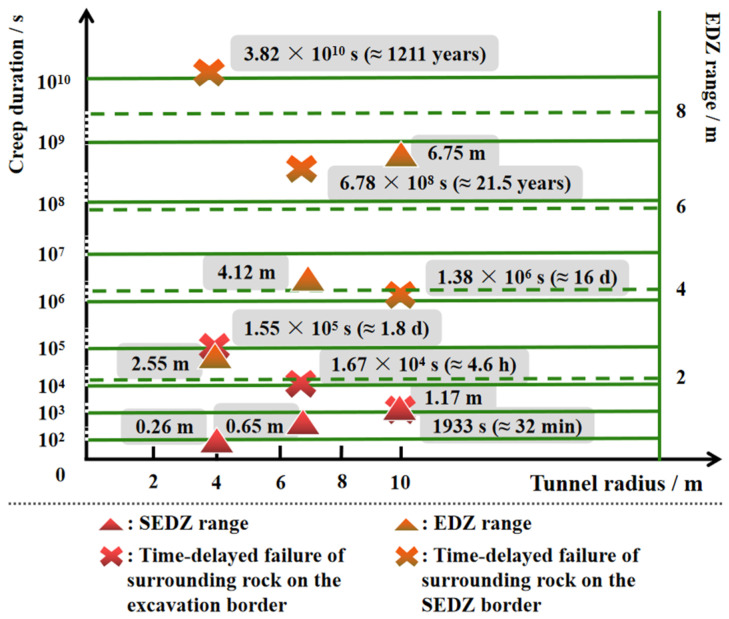
Time-delayed failure laws in the EDZ for the round tunnel cross section at the depth of 1900 m (*a* = 4, 6.5 and 10 m).

Figure 11 shows the variations of time-delayed failure duration on the condition that the radii of the opening were 4 and 10 m. Combining Figure 10, as the radius increased, both the range of WEDZ and SEDZ expanded (Figure 12), and the corresponding maximums were 5.58 and 1.17 m when the diameter was 20 m (Figure 11b). The creep durations on the boundary of the opening and SEDZ increased with reduced radius and the maximums reached 1.8 days and 1211 years (*a* = 4 m), respectively (Figure 11a). These phenomena suggest that the surrounding rock in EDZ becomes more unsteady with the increase in the excavation opening radius, particularly, the strongly damaged area in which the hance of the tunnel should be strengthened for support.

**Figure 13:**
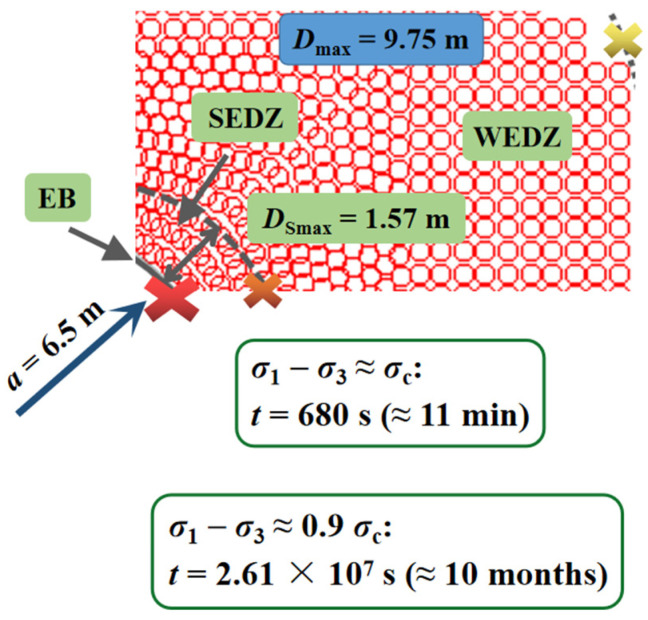
Time-delayed failure laws in the EDZ for the round tunnel cross section at the depth of 2400 m (*a* = 6.5 m).

**Figure 14:**
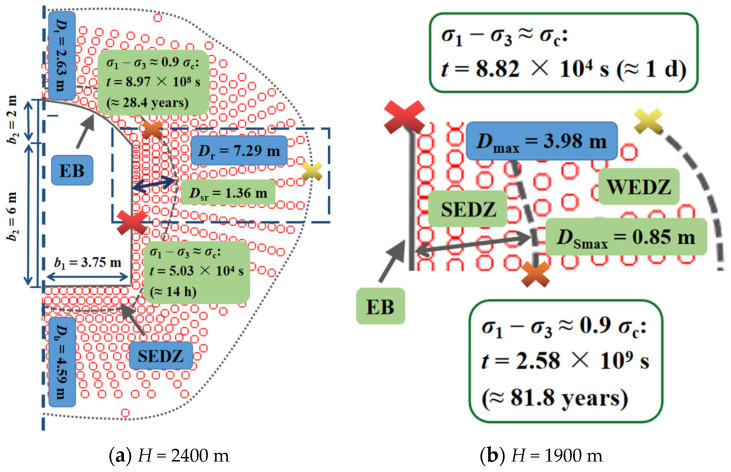
Time-delayed failure laws in the EDZ for the arched tunnel cross section at the depth of 2400 m (**a**) and 1900 m (**b**).

The numerical models for round (*a* = 6.5 m) and arched tunnel cross section (7.5 m × 8 m) under higher in situ stress at the depth of 2400 m are exhibited in Figure 13 and Figure 14a. Compared with the round tunnel model at the depth of 1900 m, the maximum scope of SEDZ increased from 0.65 to 1.57 m, and the region near EB failed in 11 min and the creep process on the SEDZ boundary lasted about 10 months, which indicate that the ground stress field makes a significant difference to the stability of the surrounding rock after excavation unloading without support, especially in the area near the excavation edge in the SEDZ at greater depth.

It can be seen in Figure 14a that for the arched tunnel model, the maximum and minimum ranges of the SEDZ were 7.29 and 2.63 m, located in the upper section of the side wall and the vault. The local stress concentration was induced by the stress redistribution for the surrounding rock around the opening after excavation unloading, causing the obvious scope difference of the damage zone. Furthermore, although the geometric dimension was similar to the case of the round cross section (*a* = 4 m) (Figure 11a and Figure 14b), both the apparently shorter time-delayed failure durations on the border of excavation and SEDZ, and wider damaged range of surrounding rock were exhibited under the effect of an irregular shape for the excavation opening section, suggesting that the cross-section shape of the deep tunnel also significantly affects the time-delayed failure of the surrounding rock and the high stress concentration area needs enough reinforcement in the SEDZ.

## 5. Concluding Remarks

In order to clarify the initiation mechanism of time-delayed rockburst and time-delayed failure characteristics for surrounding rock in a deep tunnel after excavation unloading without support, the theoretical and numerical approaches were combined for investigations from the time (creep duration) and space (damaged region) perspectives. The main conclusions of this study are as follows:

1. Due to the high damage accumulation by the developed crack evolution during the creep inside the surrounding rock, the time-delayed rockburst is easy to trigger within a short time, if the valid support for the excavation surface of the deep tunnel is not set promptly after excavation unloading.
2. The surrounding rock in the plastic state area presents more instability than that in the elastic status zone. The surrounding rock on the excavation border failed in 2.7 h and with the growing distance from the center of tunnel section, the creep duration reached three years on the plastic area boundary until the stress field achieved balance as the distance reached infinite.
3. The significant increasing tendency was exhibited by delayed failure duration when the tunnel excavation radius was 6.5 m at the depth of 1900 m, ranging from the excavation surface (4.6 h) to the interface (21.5 years) between the SEDZ and WEDZ. The surrounding rock in the SEDZ of which the maximum scope reached 0.65 m was more unsteady than that in the WEDZ, where the area close to the excavation edge most easily induced the brittle creep failure in a brief period.
4. Both the increasing radius of the tunnel cross section ranging from 4 to 10 m and the higher in situ stresses around the opening at depth (2400 m) accelerated the time-delayed failure of the surrounding rock in the SEDZ. The local high stress concentration area formed by the irregular cross-section shape resulted in the expansion of the damage zone and the decrease in the time-delayed failure duration.

This study further reflects the inextricable link between creep behavior and time-delayed failure for the surrounding rock after excavation unloading in a deep tunnel, and for the more efficient prevention of time-delayed rockburst. As an extension to this work, detailed and exploratory research regarding the influencing mechanism of timing and function of support on deep surrounding rock will be conducted in the future.

## Figures and Tables

**Table 1:** Mechanical parameters for Jinping marble on the macro- and micro-scales.

Parameters	Specific Value
Short-term strength (*σ*c/MPa)	105
Elastic modulus (*E*/GPa)	25.3
Internal friction angle (*φ*/°)	22.7
Cohesion (*c*/MPa)	23.9
Poisson’s ratio *ν*	0.3
Initial damage *D*_0_	0.048
Fracture toughness (*K*_IC_/MPa m^1/2^)	1.61
Index of stress corrosion *n*	57
Characteristic crack velocity (*v*/m s^−^^1^)	0.16
Initial crack size (*b*/m)	0.00305
Friction coefficient *μ*	0.51
Microcrack angle (*ψ*/°)	45
Constant *β*	0.32
Material constant *m*	1
Material constant *ε*_0_	0.015
Initial equilibrium crack length (*l*_0_/m)	0.0038

## Data Availability

The data presented in this study are available on request from the corresponding author.
